# An Exploratory Study on Subjective Perceptions of Happiness From India

**DOI:** 10.3389/fpsyg.2022.823496

**Published:** 2022-02-03

**Authors:** Kamlesh Singh, Shilpa Bandyopadhyay, Gaurav Saxena

**Affiliations:** Department of Humanities and Social Sciences, Indian Institute of Technology Delhi, New Delhi, India

**Keywords:** happiness, happiness determinants, Indian context, qualitative, South Asia, wellbeing

## Abstract

The present study aimed at understanding the subjective perception of happiness in a sample of Indian participants from diverse socio-economic backgrounds. Using convenience sampling, individual interviews were conducted with 60 participants aged between 19 to 73 years (M Age = 40 years). This study employed reflexive thematic analysis to analyse the written transcripts. Nine themes were generated which captured the essence of happiness for Indians—*Feelings and Expressions of Happiness; Human Ties and Happiness* which encompassed four sub-themes—*family bond, the company one keeps, the pandemic and social disengagement*, and *the nation, society and happiness; Satisfaction with Material Needs and Resources; Lifestyle and Health; Work and Play; Accomplishment and Appreciation; Nature Connectedness; Religious and Spiritual beliefs;* and *Happiness as a Personal responsibility—Role of Positive Personality Traits*. These findings revealed our respondent's multidimensional conceptualization of happiness, and adds to the growing body of happiness literature from the South Asian context.

## Introduction

Happiness is a complex, elusive and diverse concept because of the multitude of meanings that people attach to it (Oishi et al., 2013; Trinh and Khanh, 2019). Although it is extensively researched, psychologists still grapple with developing a universal definition of happiness. It is even more challenging in a multi-cultural, multi-linguistic and ecologically diverse country like India.

Culture has been recognized as shaping our perception of happiness (Joshanloo, 2014). A dominant distinction within cross-cultural studies is between western-individualist and eastern-collectivist societies (Delle Fave et al., 2016). While the Euro-American societies view happiness as individual attributes of progress and wellbeing and associate it with high arousal emotions (e.g., excitement, energetic), the eastern collectivist societies such as Japan and Korea define happiness in terms of low arousal emotions (e.g., calm, relaxation) and associate values which reflects social harmony, philanthropy, human relationships, and collective wellbeing (Ye et al., 2015).

Western notion of happiness promotes self-enhancement, fulfillment of hedonic goals by minimizing suffering, mastery over one's environment, and life satisfaction and stresses on the role of materialism in deriving happiness. Eastern cultures, by contrast, advocate for self-transcendence, and strive for eudemonic pursuits while valuing suffering, encouraging harmonious co-existence with one's environment and promoting contentment in life through spirituality and religion (Joshanloo, 2014).

Currently there is a growing body of literature on happiness across the world. Quantitative studies have identified several determinants of happiness such as quality of social relationships, achievement of goals and positive feedback, career success and progression, level of income, positive attitude, altruism, leisure and recreational activities, nature, religion and spirituality, and health and healthy lifestyle habits (Howell and Passmore, 2013; Zhang et al., 2014; Bak-Klimek et al., 2015; Azizan and Mahmud, 2018; Muthuri et al., 2020).

A review of the Indian literature on happiness shows that there is converging evidence regarding the association between happiness and strong social and familial ties (Chadda and Deb, 2013), religiosity and spirituality (Chang et al., 2018), pro-social behavior (Suar et al., 2020), goal achievement and leading a meaningful life (Sharma and Patra, 2014), and politico-economic environment (Biswas-Diener et al., 2012). In terms of personality traits, Hafen et al. (2011) found significant correlations between happiness and conscientiousness, extraversion, and emotional stability for both male and females. Apart from these happiness enablers, previous studies have also reported factors that act as happiness barriers for Indians such as witnessing the death of a loved one. Chakraborty et al. (2019) found that academic pressure, social media usage, bad past experiences, and overthinking lowers happiness among Indian college students. Additionally, lower family income and education level (Suar et al., 2020), not meeting necessities (Biswas-Diener and Diener, 2006), loneliness and lack of social support (Srivastava and Shukla, 2018), family conflict (Sandhya, 2009), and health issues (Rajeswari et al., 2005) are some other happiness inhibiting factors. Other happiness determinants include physical activity (Pengpid and Peltzer, 2019), yoga and spiritual practices such as Satsang (Singh et al., 2014a), meditation (Benada and Chowdhry, 2017), positive and optimistic thinking (Wani and Dar, 2017), being around nature (Kumar et al., 2014), sharing problems with others (Singh et al., 2014b), leisure activities (Suar et al., 2020) and having a healthy schedule which includes proper diet and sleep (Peltzer and Pengpid, 2013).

Thus far a substantial number of studies have examined the role of different factors as determinants of happiness. Although these studies have played a critical role in broadening our understanding of happiness and its factors, the existing happiness research in India has largely been quantitative with the use of self-report measures. While quantitative studies on happiness are invaluable, majority of the self-report measures, and existing theories, models and studies investigating determinants of happiness are based on researchers own conceptualization of happiness. Since these have mainly emerged from work of Euro-American scholars, these are likely to be based on western individualistic notions of happiness (Uchida et al., 2004; Delle Fave et al., 2016). Another limitation of the quantitative measurement-based studies is that they do not capture the subjective experience and points of view of people (Hardin et al., 2014; Sutton, 2021). However, being a subjective phenomenon that maybe shaped by cultural factors, the nuanced understanding of happiness in a particular cultural context necessitates an in-depth, and context-dependent exploration (Wills-Herrera et al., 2009) that can be better achieved through qualitative studies adopting a bottom-up or data-driven approach.

However, presently, majority of the qualitative literature on happiness is from Western countries (e.g., Koffman et al., 2013). We are also observing a growing number of such studies from East Asian countries (Uchida and Yuji, 2012). For instance, Lu (2001) investigated the meaning of happiness among 142 Chinese college students by asking them to write an essay on the same. Thematic analysis revealed that happiness was viewed as experiencing positive emotions, satisfaction and contentment, being in harmony with the environment and having a sense of achievement and hope. In-depth interviews with 58 young Vietnamese adults aged 20 to 31 years and subsequent thematic analysis of their interview transcripts showed that they attributed happiness to healthy social relationships, community belongingness and self-actualization (Trinh and Khanh, 2019).

Studies from other cultures such as Italy (Sotgiu, 2016) also revealed a wide diversity of factors subjectively associated with happiness. A mixed-methods study conducted on 178 Italian college students, for instance, explored their perceived determinants of happiness and unhappiness, in addition to assessing their scores on a single-item global happiness measure. Content analysis of the qualitative data revealed several happiness factors including but not limited to—family, friendship, love, health, self-actualization, success, self-knowledge, money. Another study comparing the happiness conceptualization of 57 German and 44 South African university students, as indicated in their essay on “what is happiness to you” found that German students equated happiness with autonomy, freedom, and hedonic pleasures. Whereas South African students defined happiness as a “contemplative state” including contentment, societal harmony and strong social relationships (Pflug, 2009). Another cross-cultural study investigated the perceived happiness factors among 133 Italians and 132 Cubans, aged between 11 to 74 years. Coding and categorization based on semantic similarity of the components revealed 23 categories, with only two culture-specific and 21 common factors. The culture-specific components included facing adversities and security as mentioned only by the Cubans. While, some of the perceived happiness factors common to both the cultures included health, family, money, friendship, love and work (Galati et al., 2006).

Two notable observations with regards to the existing qualitative literature on happiness is that many of them have employed college and university students who represent a limited age cohort and social class (Delle Fave et al., 2016). Secondly, there is a relative underrepresentation of South Asian countries such as India in the existing qualitative literature on happiness. Like several other non-western cultures India has a history of colonization and unique economic-political and cultural conditions. Like most Asian, African and Latin American countries, we are currently undergoing major socio-economic transformations such as globalization, urbanization, industrialization, modernization and the rapid growth of nuclear families. Besides, as has been noted in many parts of the world, it is rather difficult to find a pure form of non-western culture. In an increasingly globalized and technology-driven world most people have been exposed to western culture and lifestyle, and are influenced by both western and their traditional cultural values (Tse, 2017). Indeed, certain Indian scholars have also noted the co-existence of both individualist and collectivistic orientation, particularly among urban Indians (Jha and Singh, 2011).

The future development of India will have a substantial impact on the well-being of a large portion of the world's population, considering that we are the second-most populous country of the world. The need for more knowledge about how local people define happiness and related concepts lies at the heart of devising effective measures to improve their wellbeing (Selin and Davey, 2012). This is particularly important given India's deteriorating position in the World Happiness Report (presently 139th out of 149 countries) and growing mental health concerns. For instance, a report revealed that in 2017, one in seven Indians struggled with some mental health condition (Sagar et al., 2020).

Given this need for expanding our understanding of the happiness perspective of Indians we conducted a qualitative study using interviews with participants belonging to varied age and social groups, with a view to gain an in-depth and culture-specific understanding.

## Methods

### Participants

This study comprised a convenience sample of 60 adults (Males = 33, Females = 27) aged between 19 and 73 years (M Age = 40 years, SD= 14.66) belonging to Delhi NCR and Bhopal. Detailed socio-demographic characteristics of the participants are presented in Table 1.

**Table 1:** Socio-demographic characteristics of the participants.

**Socio-demographic characteristic**	***N***
**Age**	
19–29	15
30–39	15
40–49	12
50–59	12
> 60	6
**Education**	
No formal education	5
Primary education	5
Matriculate	2
High school	8
Graduation	19
Post-graduation or higher	21
**Marital status**	
Married	39
Single	19
Widowed	2
**Family type**	
Nuclear	40
Joint	20
**Location**	
Urban	34
Rural	26
**Religion**	
Hindu	57
Muslim	1
Christian	1
Buddhist	1
**Monthly family income (in Rs.)**	
0–30,000	16
31,000–60,000	15
61,000–90,000	4
91,000–120,000	6
121,000–150,000	7
>150,000	12
**Occupation**	
Student	4
Service	33
Self-employed	9
Retired	4
Unemployed	6
Other	4

### Materials and Procedure

This study received ethical clearance from the Institutional Ethics Committee of Indian Institute of Technology Delhi. Data was collected through semi-structured interviews (both online via Zoom meeting and face-to-face in the community) between February to March 2021. To get a diversity of views, we approached people belonging to different age ranges and socio-economic backgrounds.

Written or verbal consent (based on participant preference and their literacy status), and socio-demographic information were obtained prior to the interviews and the participants were informed that they were free to answer the interview questions either in Hindi or in English, and confidentiality and anonymity was assured. The interview schedule was also prepared in both languages. The interview questions broadly covered the meaning of happiness to the participants, their happiness facilitators and inhibitors, happy and unhappy moments of their life, their happy habits and suggestions to others for enhancing happiness. These were followed by probing questions where needed. The interviews were between 25 to 45 min. At the outset, the participants were informed that they were free to withdraw consent at any time during the interview. All the interviews were audio-recorded, and transcribed verbatim.

### Data Analysis

We used NVivo 12 for data management and analysis. Using a social constructionist framework and focusing on the semantic level of the data, we employed reflexive thematic analysis (Braun and Clarke, 2006, 2013, 2019) with an inductive or data-driven approach. The analytic process involved an active collaborative of all the three researchers to develop a richer and more nuanced understanding of the data (Braun and Clarke, 2019). We followed the six-phase process of reflexive thematic analysis as proposed by Braun and Clarke (2006), viz. data familiarization, coding, generating of themes, reviewing the themes, defining and naming them, and finally writing the analysis. These steps were followed to identify, analyze and report recurring and meaningful patterns observed in the data.

As the first step, the researchers read and re-read the transcripts multiple times to gain familiarity, while also annotating relevant extracts and noting ideas that could aid their coding in the subsequent stages. The authors read a decided number of transcripts independently, and met twice weekly on the MS Teams platform to discuss their annotations, and observations. They continued their weekly meetings throughout the analysis phase.

After gaining sufficient familiarity with the data, the next stage involved generating and assigning initial codes to meaningful extracts of the data. Once this process was repeated for all the transcripts, we initiated an in-depth evaluation by sorting and collating all the codes generated across all transcripts with the aim of identifying meaningful patterns and to produce an initial set of potential themes. In the subsequent steps, these potential themes were merged and split several times, and were scrutinized and refined. The entire analysis process involved a continuous reflective process where we actively immersed and re-immersed into the data by revisiting the transcripts and analysis phases several times, before finally developing the themes and sub-themes after a series of iterations. The themes and sub-themes were finalized only when all the authors arrived at a consensus regarding the same.

## Findings

The present analysis resulted in the development of the following nine themes and four sub-themes. The key findings of all the themes and sub-themes are summarized in Table 2.

**Table 2:** Key findings of each theme and sub-theme.

**Sr.** **No**.	**Theme**	**Key findings**
1	Feelings and Expressions of Happiness	Happiness is seen as a state of mind which is characterized by the experience of low arousal emptions such as peacefulness and contentment and high arousal bodily responses such as being excited and energized.
2	Human ties and happiness	
	*Family bond*	Strong familial relationships and wellbeing were primary determinants of happiness. Family provided emotional buffer and support to face adversities and restore happiness. Death, illness, and conflict within family impeded happiness.
	*The company one keeps*.	Being surrounded by good and supportive people provided motivational, emotional, and material support which is instrumental to happiness. They offered a non-judgmental and positive environment to confide about life stressors.
	*The pandemic and social disengagement*	Covid-19 pandemic deprived people of opportunities for physical and social interaction, inducing negative emotions and affecting people's wellbeing.
	*The nation, society and happiness*	The socio-economic-political aspects of the society regulated individual happiness.
3	Satisfaction with material needs and resources.	Money assumed different roles for people belonging to higher and lower economic strata- for those belonging to the lower socio-economic background, it was a necessity fulfill basic life necessities; and for people from higher socio-economic background, it was a means to afford luxuries.
4	Lifestyle and health	Physical and mental wellness were paramount for happiness. Good health enabled the individual to function well in other domains of life. Consequently, adoption of health promoting lifestyle practices drove happiness.
5	Work and play	Work was considered instrumental for happiness as it enabled the individual to live a comfortable life. A conducive work environment was crucial for workplace happiness. Similarly, engaging in activities for personal development and leisure also helped an individual stay happy.
6	Accomplishment and appreciation	Achievement of personal and professional goals motivated people and induced positive emotions. Acknowledgment of hard work in the form of tangible and verbal appreciation enhanced the happiness.
7	Religious and spiritual beliefs	Being spiritual and religious were important contributor to happiness. Relating to a higher entity instilled purpose, faith and helped people foster positivity and optimism despite adverse life circumstances.
8	Nature connectedness	As a peaceful and calm escape, nature offered a contemplative space to embrace positivity.
9	Happiness as a personal responsibility–The role of positive personality traits	Happiness was lauded as a personal responsibility. It was considered achievable through favorable personal traits such as positive thinking, optimism, kindness, empathy, etc.

### Feelings and Expressions of Happiness

This theme encompasses the varied emotional and bodily responses that people associate with happiness. While defining happiness, respondents recurrently described it as an inner feeling that is often expressed in terms of changed bodily expression.

Happiness was popularly characterized as a “state of mind” wherein there is “mental and emotional stability” and “soundness”. It was associated with the experience of low arousal emotions such as “peace”, “calm”, “relaxation”, “contentment” and “internal joy” that are felt from within:

For me, happiness would be… [being] content about where I am, what I am, who I am, and what am I doing… So, for me, happiness would be contentment. [It] could be just…sitting idle and you know, staring at the ceiling…the feeling that you get then. The feeling of being relaxed and chilling…the feeling of sitting idle and yet… being at peace with yourself—Participant 5, 32 years old, Farmer.

Interestingly, most considered these low arousal-based feelings to be expressed in the form of high bodily arousal which reflects “energy” and “excitement”:

When I am happy, my body becomes very active. I feel so energized…I become excited– Participant 10, 56, private-sector employee.

When happy, some participants recalled being positively motivated and in a “cheerful” and “uplifting” mood which co-occurs with certain behavioral expressions such as “laughing”, “giggling” and “smiling”:

When I am happy, I'm like feeling overjoyed…giggling and all…like I'll be smiling and I'll be energetic and I'll be…laughing—Participant 5, 32 years, Farmer.

### Human Ties and Happiness

This theme and its sub-themes capture how the meaning and experience of happiness is shaped by social interactions and human bonds:

#### Family Bond

Familial relationships were one of the most prevalent determinants of happiness. Our participants positively appraised the role of the family in providing support and acting as a buffer during hardships and stressful life events. “Sharing problems” or merely “spending time” with them was enough to move toward a happy state. Time spent with family was cherished the most and they reported extreme distress and sadness in the event of “death of a family member”, “family illness” or “conflict between family members”. While describing how these factors can negatively impact wellbeing, a participant said: “If someone [in the family] falls ill or if someone dies, like when my mother passed away, I was very upset. I became so sad that I even fell ill.” - Participant 50, 56 years, Housewife.

Besides, many of them conceded that their actions were mostly directed toward the “betterment of their family” and they recollected experiencing negative emotions such as sadness, disappointment, and frustration when they were unable to fulfill their perceived family obligations:

I will be unhappy …I fear that I will disappoint my parents if I do not achieve anything career wise. I fear that they will not be proud of me…I think if we are able to keep our loved ones happy which includes our parents, siblings… then we can keep everyone happy. Because if they are happy, then you can also be happy—Participant 60, 30 years, private employee.

#### The Company One Keeps

The quality of the social network is another factor that was perceived as influencing their level of happiness. On several occasions our participants highlighted the importance of surrounding oneself with “good” and “positive” people in order to be happy. They defined “good company” as having few [“quality over quantity”] trustworthy people in whom one can confide their problems:

I truly believe in being with positive people…that definitely elevates your feeling, and…gives you that motivation to do better… I think that's what friends are for - comforting… sharing your joys and your sorrows—Participant 41, 19 years, Student.

They also stressed the importance valuable friends who are non-judgemental, have “good intentions” and habits, “drive” and motivate them toward their goals and persistently provide emotional, and material guidance whenever sought. For Participant 7, 31 years old private employee, a good company will comprise of people: “with whom you can share without having the fear that they will judge you or you know they will make fun of your or, you know, use that knowledge to back stab you.”

#### The Pandemic and Social Disengagement

This theme pertains to the impact of the Covid-19 pandemic on the happiness of people due to disrupted social interactions. This sub-theme was relatively more prevalent in the responses of the participants belonging to the higher socio-economic background. Deprivation of physical interaction, and social isolation seems to have impacted their wellbeing. It induced feelings of restlessness, hopelessness, and helplessness. They spoke about feeling agitated while anticipating normalcy and adapted to innovative ways of being connected to friends and family through regular audio and video calls, online games, online group movie screening etc. Highlighting the impact of the social disengagement, Participant 3, a 20-year-old student remarked:

I like talking to people. If I am unable to meet someone, like in the early days of lockdown, then I get the feeling of being suffocated… Because there was no one around, I noticed a lot of changes in me as a person… it becomes impossible to go on with the day without any human interaction.

#### The Nation, Society, and Happiness

The prevailing socio-economic-political issues in the country were also mentioned by our interviewees as impacting their happiness level. They stressed that their dissatisfaction with the government's ideologies, policies and the performance of the various executive and legislative functionaries, poor economic performance, and prevalence of unfair practices like corruption, within bureaucracy was an inhibitor of their happiness:

Seeing my country in this situation where people are suffering because of unemployment, corruption… millions of youths are… unemployed, how can I be happy in such a situation? Participant 58, 50, Businessman.

Interviewees also mentioned about the negative impact of several social practices—the ones which violate the notion of equality, such as gender equality and impedes one's right to exercise freedom of choice:

…it's a societal conditioning as I was growing up, even today, I feel that generally women grow up with a sense of they are told what their duties are. Our society has not encouraged that thinking for yourself… is right. We've always been taught to put ourselves behind…everyone has a mind and they should be given the freedom to exercise it—Participant 20, 53, Teacher.

Given that societal evils and inequalities and governmental functioning are not under one's control, the participants mentioned that they tried doing something positive for society in their individual capacity and this was as a major source of happiness for many of them:

…the situation of your country…is deviating from democracy…free speech is being questioned. So, all these really makes you sad. In my individual capacity I won't be able to do anything much about it… But there are things like if you talk about this environment issues so I can do a whole lot of things at personal level, so I do that use. Public transport, use things which are recyclable, consume less energy. So, all this I can do so I feel good about it…—Participant 21, 58, Educationist.

### Satisfaction With Material Needs and Resources

Another major subjective determinant of happiness, as observed in this study, was the fulfillment of basic life necessities. While the role of “money” or “financial resources” was recognized by all, its utility was acknowledged differently by people belonging to the lower and higher economic strata. For people in the lower income group, money is the primary source for meeting necessities such as “food, safe drinking water and shelter”. They also drew attention to the “financial burden” because of “loss of jobs”, livelihood and significantly “reduced income” due to Covid-19 related lockdown.

I think everyone is unhappy because of Corona…especially every working man is sad because corona has taken back our progress by over 10 years. Take my example, I used to get full month's salary and now I only get salary for 15 days. What can we even do with so little money? My family comprises of 5–6 members. With so little money, I must take care of all of them and at the same time take care of all household expenses such as milk, water, oil etc. So, I believe that once the situation gets better, I will be happy—Participant 29, 47 years, Private-sector.

However, for people belonging to the higher income group, money was primarily seen as a means of affording luxuries and other materialistic comforts in life. Therefore, for the former it was about having sufficient finances so as to afford basic necessities for self and family (excerpt—1), while for the latter, it was about having resources that could help in leading a comfortable and luxurious life (excerpt—2):

Excerpt 1: I become unhappy when I reach home and there is no money. It is very upsetting when a person does not have any food to eat at the end of the day. He wonders that despite working hard the entire day and night, he still does not have sufficient food to eat at home…also, another source of unhappiness is when you are unable to pay the house rent…there are additional expenses associated with children's education too. If one does not have enough money to pay their fee…this causes a lot of unhappiness.—Participant 15, 30 years, Private employee, lower SES.

Excerpt 2…I have always wanted to own an amazing car and my own house. My mother and I specifically want to live in a house with a balcony. So, when I have that and I come home to find other things which I wished for, then that will be a perfect happy day for me—Participant 42, 23 years, Student, Higher SES.

### Lifestyle and Health

Physical and mental wellbeing and lifestyle practices was also found to be a pressing theme in this study. Many participants recognized the role of health [both physical and mental] on happiness. As most of them admitted, other happiness-inducing factors such as money and material things are “secondary” in comparison to the health and wellness of oneself and one's family. For Participant 39, a 38-year-old female, good health was the pre-requisite for enjoying the little things of life:

If your health is not good, how much ever good the situation is, you will not feel happy. So, if you're healthy, if you are feeling good then only you can enjoy the little things in life. If health is fine, everything is fine, and if health is not fine, nothing is fine.

Interviewees stressed that the presence of any minor or major illnesses, or merely its anticipation was enough to exponentially reduce their level of happiness and induce extreme “stress”. Especially for people belonging to the lower economic strata, illness puts additional burden because they often lack sufficient resources to afford quality treatment and medical intervention:

Recently my father was very unwell. It was very unhappy that time…I didn't know how I will be able to manage…I don't have enough money to afford the expenses…sometimes I doubt and worry if I will be able to take care of my family since I don't have money—Participant 13, 41 years, Private sector employee, LSE.

Therefore, to avoid ailments, participants were motivated to adhere to a good and healthy lifestyle. This involves following a disciplined routine that entails “sleep[ing] well, eat[ing] well”, having a “healthy diet”, and regularly engaging in health and wellness enhancing activities such as “exercise”, “yoga”, “meditation”. Participants also acknowledged the importance of “time management” in striking a proper balance between various life commitments and in practicing health-promoting behaviors. While describing his version of a “happy day”, Participant 9, a 24-year-old, a private-sector employee, highlighted how planning a schedule and sticking to it made him happy:

…[a] normal routine would be where I'm satisfied with all the things that I have planned. So, I plan things. Basically, I like to plan certain things like my normal routine. I love to plan. That so and so things I need to do. If that goes all well, then I'm happy…

### Work and Play

Broadly, this theme highlights the importance of both work and recreation in the lives of our participants.

While they considered work to be a major facilitator of their happiness, it took on a different meaning for people belonging to the higher and lower income group. For people in the higher economic strata, work gave them happiness when their job was in alignment with their interests and passion. They also expressed the importance of a “conducive work environment” which reinforces their potential, where there is harmony and good relationship between co-workers along with the availability of opportunities for personal and professional growth:

…my work… for me, it's really important that every day I learn something new there…So if I have done something or I've learned something new, I feel extremely satisfied and happy. Participant 39, 38 years, Doctor.

People in the lower income bracket, on the other hand, primarily focused on the need to work diligently and to have a secure job to ensure a stable source of livelihood:

A person should work hard for a secure job. When they will get paid for their work, have a regular source of income they will feel happy from within because then they will be able to afford things they want - Participant 11, 38 years, Private sector employee.

If work was highlighted as important, so was play. Devoting time to “hobbies” or recreational activities such as “dancing, singing, painting”, “cooking”, “writing poetry”, “reading”, “gaming”, “traveling” or other artistic or creative endeavors enabled the respondents to “take a break” from the stress and monotony in their lives, and “refreshed” them by providing the necessary “me time”. Both work and play were seen as effective distractors during times of distress:

Your hobbies will play an important role [in making you happy] …if I continuously watch TV, then my mood changes, I become happy…Some people say, okay I like playing games, some may say I prefer TV or internet surfing helps me be happy. So, it depends…person to person – Participant 25, 62 years old, Retired.

### Accomplishment and Appreciation

This theme encapsulates the experience of happiness which stems from accomplishment and subsequent positive appraisal in the form of appreciation by others and self. Our participants advocated the importance of “hard work” in achieving one's goals and the importance of demonstrating resilience in the face of adversity. Participants saw the achievement of “little” or “big” goals as happiness, be it professionally or personally. Professionally, they derived happiness from achieving “work-related targets”, career growth or promotion, or through overcoming a challenge independently. While participants admitted the inability to achieve goals as a major source of distress and frustration, they worked consistently, often with more vigor, to reach their goals. “Seeking help”, “taking a pause”, becoming “self-aware of limitations”, “accepting mistakes”, “sharing” about the problem with loved ones and having a positive mindset helped them stay motivated in the face of failure in accomplishing their mission:

When I am stuck with a problem and when I am really unhappy…if I talk with someone that would solve my problem, then I would definitely want to bring up a conversation with my family members or with my wife or someone, you know, [for] a solution for the problem—Participant 8, 33 years, Private sector employee.

A source of happiness related to accomplishment was the appreciation received for it. Participants lauded the influence of “positive feedback” or “appreciation” in the form of material tokens or “praises”, on their level of happiness. These not only validated their effort but also motivated them to improve their personal and professional performance:

When I started playing guitar, I somehow felt that I would not be able to do it because it damages hands a lot. I felt that this is very difficult, but I took it as a challenge. So, when I did it, when I finally started playing it, people around me started appreciating me a lot. They were surprised that I learned how to play the guitar so quickly. That gave me energy…it feels good that I can live up to my potential. I can overcome my barriers. I like it when people get surprised…I get a boost and I crave for that… I want people to appreciate me even more… This makes me very happy—Participant 3, 20 years, Student.

Besides, in the case of some of our participants, an “absence of [anticipated] appreciation” gave rise to feelings of unhappiness. In such situations, they adopted the strategy of self-appreciation to derive happiness. Self-appreciation can be in the form of “self-care” by “treating oneself” or by simply feeling “content”, “confident”, and “reassuring” themselves of their worth. Participant 7, 31 years, explained how he incentivized and celebrated his wins by himself:

I give a treat to myself… like going out for a movie or getting something which I wanted for a long time but was postponing it like a gift, a cool tech…a mobile or something which I was postponing.

### Religious and Spiritual Beliefs

This theme pertains to the role of religion and spirituality in helping our participants lead a happy life:

I feel happy when I worship my Guru… if for any reason, I fail to worship my Guru, I will be extremely unhappy. The more I devote myself to my Guru, the happier I will be, this is what I believe…thinking about Guru makes me happy…and that helps me navigate through tough life situations – Participant 45, 42 years, Businesswoman.

Many of them reported routine engagement in activities such as “prayers”, “reading religious texts”, singing “*bhajans”* (or religious songs) as a way of advancing their spiritual and religious beliefs. These practices not only helped them stay connected to (their) ancestors but also guided them toward a good life—by teaching them how to be a “nice person” who is considerate to the feelings of others:

I think it just makes me keep connected to myself as well as the ancestry…we've always been taught of being proud of who we are, where we come from, and there's a certain level of duty that we also owe toward our ancestors as well as [other] human beings. And I think being connected to spirituality makes me a better person—Participant 24, 27 years, Private sector employee.

Interviewees pervasively associated their religious and spiritual practices with “faith”. Faith was construed as a multifaceted construct that instills “positivity” and “hope” for a bright future, motivates one to face and overcome challenges and provides opportunities for introspection and self-discovery. They recalled feelings of “positivity” and “peace” when practicing faith and emphasized the need to be grateful to God for whatever life offers, including the hardships. For instance, for Participant 44, a 46-year-old homemaker, spending even half an hour inside the temple adjacent to her house provided her with a sense of positivity and respite from household problems and tensions. She described her feelings while visiting the temple as:

I mean it feels like there's only God, and nothing beyond God for me. I have worked at home all my life but the level of satisfaction and peace that I get there [in a temple], is unmatched. Even if I spend half hour, I feel like I am in another world altogether. No matter how many problems are there at home, how much tension is there, if I go to the temple, I get immense positivity. This feeling is impossible to explain and I can never get it from anywhere else.

### Nature Connectedness

This theme reflects the influence of nature on happiness. Participants viewed nature as an effective escape from several “negative” elements in their life. Apart from popularly acknowledging “scenic beauty”, interviewees noted that being around nature offered a calm and relaxing environment that helped them reflect and contemplate on the good things in life. Observing the various elements of nature such as sunlight, green plants and trees, animals, and birds were accredited to inducing a sense of positivity, freshness, and energy. For instance, speaking of the happiness that she derived from nurturing and witnessing the growth of plants, Participant-51 remarked:

It feels like heaven… You can find happiness in plants and trees. It feels like they talk to you. It feels so good when you water them. They grow faster when you crease them and nurture them with love—Participant 51, 70 years, Retired.

Speaking more generally of the positive impact of nature, another participant emphasized that being around nature acted as a respite from the negative things happening around her, allowed her to think positively and made her happy:

…there are many negative things that happen around you every day, it is common for everyone, but when I am around nature, I'm able to get those negativities out of my mind… And I am able to think positive…I'll be happy that… I have the nature in front of me—Participant 41, 19, Student.

### Happiness as a Personal Responsibility—The Role of Positive Personality Traits

This theme embodies the idea that finding joy and happiness is a personal responsibility. While external factors can certainly dictate the level of happiness or cause unhappiness, rather than expecting society and its members to make them happy, respondents emphasized that an individual should take command and try to find happiness in their lives—

Don't let your emotions be dependent on somebody else or don't let somebody dictate the way you're supposed to enjoy yourself. You are in charge of your emotions and if you want to be happy, you're the only person who can make yourself [happy]. If you let your surroundings dictate your emotions to you, then I don't think you can ever be as happy as you want to be, whereas [if] you are in charge of whatever emotion you want to feel, then nothing can stop you from having a happy day or achieving the happiness that you desire—Participant 1, 22 years, Student.

This can be done through “positive thinking” or by having a “positive perspective” in life, “being satisfied with the available resources” and “having realistic wishes”, “building emotional capacity” to face adversities and the like. Interviewees stressed that certain personal qualities or personality traits can help an individual stay happy. According to them, a happy person will be “optimistic”, “extraverted”, “have high self-esteem”, “self-motivated”, be “self-aware”, have “moral consciousness”, be “determined”, “kind”, “empathic”, and have “humor”, “patience” and tolerance. Participants also recurrently stressed the importance of “living in the present moment” for one's happiness:

A person will be happy if he lives in the present moment. Present moment is good for any individual. In a day, all 24 h are not bad, only few moments are bad…everything is in the present. Future makes a person scared and gives a lot of suffering. It is not healthy to think about the future. People who are positive are happy, and only the present moment is positive—Participant 43, 54 years, Businessman.

## Discussion

The present study was a qualitative inquiry into the conceptualization of happiness in the Indian context. We identified nine themes that captures the essence of happiness—*Feelings and Expressions of Happiness; Human Ties and Happiness* encompassing four sub-themes- *family bond, the company one keeps, the pandemic and social disengagement*, and *the nation, society and happiness; Satisfaction with Material Needs and Resources; Lifestyle and Health; Work and Play; Accomplishment and Appreciation; Nature Connectedness; Religious and Spiritual beliefs;* and *Happiness as a Personal responsibility—The Role of Positive Personality Traits*.

Our findings revealed that happiness was subjectively associated with felt emotions of peacefulness, calmness and contentment, that have been characterized as “low arousal emotions” in the literature (Lim, 2016). While western research generally associates happiness with high arousal emotions such as elation and euphoria (Lu and Gilmour, 2004; Lim, 2016), our findings correspond with research from the eastern cultures where happiness was associated with the experience of subtle and positive valence emotions like relaxation, calmness and contentment (Uchida and Kitayama, 2009; Joshanloo, 2014). Moreover, happiness was not reported as an isolated internal experience but was accompanied by pleasant bodily changes that reflect a state of being positively motivated and energized, indicating that the experience and expression of happiness may not necessarily always complement each other. However, the qualitative reports of the arousal level associated with happiness requires careful examination by future experimental and quantitative studies.

Consistent with a large body of happiness research conducted both in the east (e.g.,: Datu and Valdez, 2012) and the west (e.g.,: Koffman et al., 2013), this study revealed the subjective importance of social interactions and meaningful human connections in driving happiness. Here, familial happiness and wellbeing had the most prominent reciprocal influence on personal happiness. Similar to what has been documented (Bak-Klimek et al., 2015; Muthuri et al., 2020), family progress, spending time with family and sharing problems increased happiness; and conflicts within the family, loss of loved ones and fear of disappointing family were identified as barriers to happiness. Support from family acts as an emotional buffer during troubling times.

Such buffer also came from friends, acquaintances and colleagues who provided motivation and positive environment for personal growth. Our findings revealed the importance that individuals attach to surrounding themselves with good and positive company that further elevates their mood and helps them stay happy. Existing quantitative findings evidences a strong positive association between friendship and happiness (Demir et al., 2011).

While human interaction was crucial for happiness, social disengagement due to the Covid-19 lockdown was identified as a subjective barrier, especially for people belonging to the middle or upper economic strata. Indeed, studies have shown that isolation and quarantine due to Covid-19 and constant worry about the wellbeing of self and family are linked to increased depression and anxiety symptoms (Grover et al., 2020; Saxena and Garg, 2020).

Moving on, the present analysis also showed that happiness was not narrowly confined to one's immediate surrounding, but extended to other members of society and the nation. For instance, the socio-politico-economic situation of the country regulated one's level of happiness. Consistent with the previous literature (see Veenhoven, 2000, 2015), social issues such as the curtailment of freedom, equality and discrimination emerged as factors impeding happiness. Similarly, in terms of the political and economic factors (as noted by Biswas-Diener et al., 2012), dissatisfaction with government functionaries and political malpractices such as corruption and economic factors such as unemployment and poverty were found to be other happiness barriers.

Our findings also showed the significance that was attached to having sufficient resources for meeting basic life needs. Research on the association between money and happiness has been conflicting (Mogilner, 2010). For example, Lakshmanasamy (2010) showed that income influenced happiness but its role diminishes after income raises beyond a certain threshold. Our study lends support to this finding, especially because money assumed different roles for people from different economic standings. Therefore, while having money to afford necessities such as food, shelter for self and family was sufficient to make people with economically weak background happy, those from affluent backgrounds saw money as the means to material luxuries for enhancing happiness. This finding also relates to Maslow's (1943) hierarchy of need model. The source(s) of happiness were different because respondents were at different hierarchical levels; striving for different needs. Consequently, the availability of monetary resources assumed a primary role for the lower economic group. This was further exemplified by the financial burden on them due to the Covid-19 lockdown which caused a dramatic loss of livelihood (Estupinan and Sharma, 2020) and was cited as a happiness inhibitor.

However, our participants also agreed that the presence of abundance of resources are of no use if they or their loved ones are not healthy—physically and mentally. In fact, a popular saying in the Indian context is *pahla sukh nirogi kaya* which means the first and foremost happiness in life is a healthy body (Modi, 2017). In the context of health and happiness, participants were inclined toward leading a structured life guided by healthy lifestyle practices which includes healthy eating, proper sleep, regular physical activity in the form of exercise and yoga and meditation. These practices have positive associations with improved mental health and happiness (Pengpid and Peltzer, 2019; Muthuri et al., 2020; Sahni et al., 2021).

While having a stable job was an important determinant of happiness for individuals with relatively low income, working productively in a conducive and supportive environment, which aligns with one's interests, and reinforces professional development, was more critical for the higher-income group. Pursuing one's hobbies and engaging in recreational activities was a happiness booster for most of our respondents regardless of their socio-economic background, as evidenced in the existing literature (Lu and Argyle, 1994; Muthuri et al., 2020).

Another theme was the influence of appreciation in inducing happiness. This finding aligns with research which shows that in the workplace, verbal or tangible appreciation reinforces thriving and vigor, job productivity and satisfaction (Fisher, 2010; Bakker et al., 2014; Adnan Bataineh, 2019). Similarly, accomplishment of personal and professional goals also emerged as dominant sources of happiness. One of the ways through which participants celebrated their wins was by indulging in several self-care activities which induced low arousal happy emotions. While goal achievement has widely been documented as a happiness facilitator (e.g.,: Suar et al., 2020), failure was reported as a major inhibitor of happiness. To cope up with failures, our participants listed several strategies such as seeking help, building acceptance, self-reflection and talking to loved ones which helped manage stress and promote happiness to some extent.

Being affiliated with nature was also a catalyst of happiness. A sizable amount of research has elaborated on our finding of nature's contribution to increasing happiness and improving mental health (Kumar et al., 2014). Along with nature, our participants found comfort in engaging in religious and spiritual activities. Apart from the fact that Indian culture is deeply rooted in and recognized for its religious and spiritual climate (Singh et al., 2019), a vast body of eastern and western research (like Koffman et al., 2013; Singh et al., 2019) has highlighted the association between religiosity and spirituality and happiness.

Lastly, happiness was emphasized as a personal responsibility. For our participants, some behavioral, attitudinal and personality traits can aid the process of keeping oneself happy. The qualities listed by them included positive and optimistic thinking, building strong relationships, kindness, being empathetic, altruistic, self-motivated and self-aware, having realistic wishes, having a strong sense of moral consciousness and emotional capacity (Hafen et al., 2011; Datu and Valdez, 2012; Holder et al., 2012; Black and Kern, 2020; Suar et al., 2020).

Broadly, happiness was seen as a multidimensional construct with inside-out and outside-in dimensions. The Inside-out aspect was characterized by traits such as optimism, empathy, altruism, spirituality that complemented the individual's innate experience of calmness, peace, contentment, symbolizing happiness. The outside-in, on the other hand, comprised elements external to the individual that enhanced happiness. In our study, these involved meaningful social bonds, professional and personal growth, receiving admiration, good health and naturalistic environment. Happiness was viewed as the harmonious interplay of these elements. As revealed in this study, the facilitating factors interact with one another at the face of adversities to restore happiness. Therefore, an individual's recovery from illness is supplemented with feelings of hope and optimism instilled by supportive family, friends and religious and spiritual faith.

Our conception captures the essence of dominant happiness theories such as PERMA (Positive emotions, Engagement, Relationship, Meaning, Accomplishment, Seligman, 2011), Subjective Wellbeing (positive affect without negative affect and life satisfaction, Diener, 2000), Self-determination theory (fulfillment of three basic psychological needs: autonomy, competence, relatedness, Ryan and Deci, 2000) and Adaptation-level theory (happiness a stable state despite life adversities, Brickman and Campbell, 1971). It also complements the eastern conception of happiness including inner harmony, togetherness, discovery of true self, selflessness and self-transcendence. In this study, the subjective conception of happiness among Indians was an amalgamation of both eastern and western tenets, with a slight inclination toward the eastern emphasis on social flourishing and focus on others. Surprisingly, despite large body of literature evidencing the role of spirituality, religiosity and nature in increasing happiness (Hackney and Sanders, 2003; Capaldi et al., 2014), these are not recognized as primary domains of happiness in the dominant theories and models of happiness. Future research can broaden the scope of mainstream theories to give them greater representation.

Although to the best of our knowledge, the present study is the first to illuminate the happiness perspective of Indians, the study findings must be considered in the backdrop of its limitations. Since we collected data only from a north Indian and a central Indian state, there is an absence of perspective from the other parts of India. Future studies may be conducted using a more diverse sample including participants across different Indian states.

## Conclusion

The present qualitative exploration aimed at understanding the meaning of happiness to Indians. Reflexive thematic analysis, using an inductive approach resulted in the generation of nine themes and four sub-themes which revealed their multifaceted conceptualization of happiness. Apart from filling an important gap in the literature, i.e., representing the happiness perspective of Indians, the present findings also have implications for future intervention studies as well as for various stakeholders such as policy makers, counselors, educators and employers.

## Data Availability Statement

The raw data supporting the conclusions of this article will be made available by the authors, without undue reservation.

## Ethics Statement

The studies involving human participants were reviewed and approved by IIT Delhi Institute Ethics Committee. The patients/participants provided their written informed consent to participate in this study.

## Author Contributions

KS conceptualized the study. GS collected data. KS, SB, and GS were involved in data analysis. All authors worked on the first and final version of the manuscript.

## Funding

This research was part of a project that was funded by the Happiness Ministry, Madhya Pradesh Government [Grant number: RP03926]. SB is a UGC-SRF and would like to thank UGC for sponsoring her doctoral fellowship.

## Conflict of Interest

The authors declare that the research was conducted in the absence of any commercial or financial relationships that could be construed as a potential conflict of interest.

## Publisher's Note

All claims expressed in this article are solely those of the authors and do not necessarily represent those of their affiliated organizations, or those of the publisher, the editors and the reviewers. Any product that may be evaluated in this article, or claim that may be made by its manufacturer, is not guaranteed or endorsed by the publisher.

## References

[B1] Adnan Bataineh K. (2019). Impact of work-life balance, happiness at work, on employee performance. Int. Bus. Res. 12, 99–112. 10.5539/ibr.v12n2p99

[B2] Azizan N. H. Mahmud Z. (2018). Determinants of Subjective Well-Being: A Systematic Review. Environ Behav. Proc. J. 3, 135. 10.21834/e-bpj.v3i7.1228

[B3] Bakker A. B. Demerouti E. Sanz-Vergel A. I. (2014). Burnout and work engagement: The JD–R approach. Annu. Rev. Organ. Psychol. Organ. Behav. 10.1146/annurev-orgpsych-031413-091235

[B4] Bak-Klimek A. Karatzias T. Elliott L. Maclean R. (2015). The determinants of well-being among international economic immigrants: A systematic literature review and meta-analysis. Appl. Res. Qual. Life. 10, 161-188. 10.1007/s11482-013-9297-8

[B5] Benada N. Chowdhry R. (2017). A correlational study of happiness, resilience and mindfulness among nursing student. Indian J. Posit. Psychol. 8, 105–107. Available online at: http://www.i-scholar.in/index.php/ijpp/article/view/157077

[B6] Biswas-Diener R. Diener E. (2006). The subjective well-being of the homeless, and lessons for happiness. Soc. Indic. Res., 76, 185–205. 10.1007/s11205-005-8671-9

[B7] Biswas-Diener R. Tay L. Diener E. (2012). Happiness in India. In Happiness across cultures. Springer. p. 13–25. 10.1007/978-94-007-2700-7_2

[B8] Black B. A. Kern M. L. (2020). A qualitative exploration of individual differences in wellbeing for highly sensitive individuals. Palgrave Commun., 6, 1–11. 10.1057/s41599-020-0482-8

[B9] Braun V. Clarke V. (2006). Using thematic analysis in psychology. Qual. Res. Psychol., 3, 77–101. 10.1191/1478088706qp063oa32100154

[B10] Braun V. Clarke V. (2013). Successful qualitative research: A practical guide for beginners. Sage. Available online at: https://uk.sagepub.com/en-gb/eur/successful-qualitative-research/book233059

[B11] Braun V. Clarke V. (2019). Reflecting on reflexive thematic analysis. Qual. Res. Sport. Exerc. Health., 11, 589-597. 10.1080/2159676X.2019.1628806

[B12] Brickman P. D. Campbell D. T. (1971). Hedonic relativism and planning the good society. In Adaptation-level theory. Appleby M. H. (Ed.). New York: Academic Press. p. 287–302.

[B13] Capaldi C. A. Dopko R. L. Zelenski J. M. (2014). The relationship between nature connectedness and happiness: A meta-analysis. Front. Psychol. 5, 976. 10.3389/fpsyg.2014.00976 25249992 PMC4157607

[B14] Chadda R. K. Deb K. S. (2013). Indian family systems, collectivistic society and psychotherapy. Indian J. Psychol. 55, S299. https://www.indianjpsychiatry.org/text.asp?2013/55/6/299/105555 10.4103/0019-5545.105555 23858272 PMC3705700

[B15] Chakraborty B. Maji S. Sen A. Mallik I. Baidya S. Dwibedi E. (2019). A study on happiness and related factors among Indian college students. Quant. Econ. J. 17, 215–236. 10.1007/s40953-018-0125-8

[B16] Chang E. C. Yu T. Lee J. Kamble S. V. Batterbee C. N.-H. Stam K. R.. (2018). Understanding the association between spirituality, religiosity, and feelings of happiness and sadness among HIV-positive Indian adults: Examining stress-related growth as a mediator. J. Relig. Health. 57, 1052–1061. 10.1007/s10943-017-0540-8 29302854

[B17] Datu J. A. D. Valdez J. P. M. (2012). Exploring Filipino adolescents' conception of happiness. Int J Res. Stud. Psychol. 1, 21–29. 10.5861/ijrsp.2012.251

[B18] Delle Fave A. Brdar I. Wissing M. P. Araujo U. Castro Solano A. Freire T.. (2016). Lay definitions of happiness across nations: The primacy of inner harmony and relational connectedness. Front. Psychol. 7, 30. 10.3389/fpsyg.2016.00030 26858677 PMC4726797

[B19] Demir M. Özen A. Dogan A. Bilyk N. A. Tyrell F. A. (2011). I matter to my friend, therefore I am happy: Friendship, mattering, and happiness. J. Happiness. Stud. 12, 983–1005. 10.1007/s10902-010-9240-8

[B20] Diener E. (2000). Subjective well-being: The science of happiness and a proposal for a national index. Am. Psychol. 55, 34. 10.1037/0003-066X.55.1.34 11392863

[B21] Estupinan X. Sharma M. (2020). Job and wage losses in informal sector due to the COVID-19 lockdown measures in India. Available online at: https://papers.ssrn.com/abstract=3680379

[B22] Fisher C. D. (2010). Happiness at work. Nt. J. Manag. Rev. 12, 384–412. 10.1111/j.1468-2370.2009.00270.x

[B23] Galati D. Manzano M. Sotgiu I. (2006). The subjective components of happiness and their attainment: A cross-cultural comparison between Italy and Cuba. Soc. Sci. Inf. 45, 601–630. 10.1177/0539018406069594

[B24] Grover S. Sahoo S. Mehra A. Avasthi A. Tripathi A. Subramanyan A.. (2020). Psychological impact of COVID-19 lockdown: An online survey from India. Indian J. Psychiatry. 62, 354. 10.4103/psychiatry.IndianJPsychiatry_427_20 33165368 PMC7597717

[B25] Hackney C. H. Sanders G. S. (2003). Religiosity and mental health: A meta-analysis of recent studies. J Sci Study Relig. 42, 43–55. 10.1111/1468-5906.t01-1-00160

[B26] Hafen C. A. Singh K. Laursen B. (2011). The happy personality in India: The role of emotional intelligence. J. Happiness. Stud. 12, 807–817. 10.1007/s10902-010-9228-421942699

[B27] Hardin E. E. Robitschek C. Flores L. Y. Navarro R. L. Ashton M. W. (2014). The cultural lens approach to evaluating cultural validity of psychological theory. Am. Psychol. 69, 656–668. 10.1037/a0036532 24841337

[B28] Holder M. D. Coleman B. Singh K. (2012). Temperament and happiness in children in India. J. Happiness. Stud. 13, 261–274. 10.1007/s10902-011-9262-x

[B29] Howell A. J. Passmore H.-A. (2013). The nature of happiness: Nature affiliation and mental well-being. In Mental well-being. Springer. p. 231–257. 10.1007/978-94-007-5195-8_11

[B30] Jha S. D. Singh K. (2011). An analysis of individualism-collectivism across Northern India. J. Indian Acad. Appl. Psychol. 37, 149–156.

[B31] Joshanloo M. (2014). Eastern conceptualizations of happiness: Fundamental differences with western views. J. Happiness. Stud. 15, 475–493. 10.1007/s10902-013-9431-1

[B32] Koffman J. Morgan M. Edmonds P. Speck P. Siegert R. Higginson I. J. (2013). Meanings of happiness among two ethnic groups living with advanced cancer in south London: a qualitative study. Psycho-Oncology. 22, 1096–1103. 10.1002/pon.3108 22639245

[B33] Kumar R. Lal R. Bansal Y. Sethi K. V. K. (2014). Relationship between connectedness to nature and subjective well-being. Indian J. Psychol. 4, 59–63. http://www.napsindia.org/wp-content/uploads/2017/05/Rajesh-Kumar-08-min.pdf

[B34] Lakshmanasamy T. (2010). Are you satisfied with your income? The economics of happiness in India. Quant. Econ. J. 115–141. https://ideas.repec.org/a/jqe/jqenew/v8y2010i2p115-141.html

[B35] Lim N. (2016). Cultural differences in emotion: differences in emotional arousal level between the East and the West. Integr. Med. Res. 5, 105–109. 10.1016/j.imr.2016.03.004 28462104 PMC5381435

[B36] Lu L. (2001). Understanding happiness: A look into the Chinese folk psychology. J. Happiness. Stud. 2, 407–432. 10.1023/A:1013944228205

[B37] Lu L. Argyle M. (1994). Leisure satisfaction and happiness as a function of leisure activity. Gaoxiong yi xue ke xue za zhi= The Kaohsiung journal of medical sciences. 10, 89–96. https://pubmed.ncbi.nlm.nih.gov/8176776/8176776

[B38] Lu L. Gilmour R. (2004). Culture and conceptions of happiness: Individual oriented and social oriented SWB. J. Happiness. Stud. 5, 269–291. 10.1007/s10902-004-8789-5

[B39] Maslow A. H. (1943). A theory of human motivation. Psychol Rev. 50, 370. 10.1037/h0054346

[B40] Modi I. (2017). Leisure, health and wellbeing: The ultimate quest of humanity. In Leisure, health and well-being. Springer. p. 273–279. 10.1007/978-3-319-33257-4_20

[B41] Mogilner C. (2010). The pursuit of happiness: Time, money, and social connection. Psychol. Sci. 21, 1348–1354. 10.1177/0956797610380696 20732902

[B42] Muthuri R. N. D. K. Senkubuge F. Hongoro C. (2020). Determinants of happiness among healthcare professionals between 2009 and 2019: a systematic review. Humanit. Soc. Sci. Commun. 7, 1–14. 10.1057/s41599-020-00592-x

[B43] Oishi S. Graham J. Kesebir S. Galinha I. C. (2013). Concepts of happiness across time and cultures. Pers. Soc. Psychol. Bull. 39, 559–577. 10.1177/0146167213480042 23599280

[B44] Peltzer K. Pengpid S. (2013). Subjective happiness and health behavior among a sample of university students in India. Soc. Behav. Pers. 41, 1045–1056. 10.2224/sbp.2013.41.6.1045

[B45] Pengpid S. Peltzer K. (2019). Sedentary behaviour, physical activity and life satisfaction, happiness and perceived health status in university students from 24 countries. Int. J. Environ. Res., 16, 2084. 10.3390/ijerph16122084 31200427 PMC6617209

[B46] Pflug J. (2009). Folk theories of happiness: A cross-cultural comparison of conceptions of happiness in Germany and South Africa. Soc. Indic. Res. 92, 551–563. 10.1007/s11205-008-9306-8

[B47] Rajeswari R. Muniyandi M. Balasubramanian R. Narayanan P. (2005). Perceptions of tuberculosis patients about their physical, mental and social well-being: a field report from south India. Soc. Sci. Med. 60, 1845–1853. 10.1016/j.socscimed.2004.08.024 15686814

[B48] Ryan R. M. Deci E. L. (2000). Self-determination theory and the facilitation of intrinsic motivation, social development, well-being. Am. Psychol. 55, 68. 10.1037/0003-066X.55.1.68 11392867

[B49] Sagar R. Dandona R. Gururaj G. Dhaliwal R. Singh A. Ferrari A.. (2020). The burden of mental disorders across the states of India: the Global Burden of Disease Study 1990–2017. Lancet Psychiatry. 7, 148–161. 10.1016/S2215-0366(19)30475-4 31879245 PMC7029418

[B50] Sahni P. S. Singh K. Sharma N. Garg R. (2021). Yoga an effective strategy for self-management of stress-related problems and wellbeing during COVID19 lockdown: a cross-sectional study. PloS ONE. 16, e0245214. 10.1371/journal.pone.0245214 33566848 PMC7875402

[B51] Sandhya S. (2009). The social context of marital happiness in urban Indian couples: Interplay of intimacy and conflict. J Marital Fam Ther. 35, 74–96. 10.1111/j.1752-0606.2008.00103.x 19161585

[B52] Saxena G. Garg M. (2020). Comparison of Psychological Impact of Covid-19 Pandemic among Frontline Healthcare Professionals in a Tertiary Care Hospital of North India. J Int Med Res. 6, 1. Available online at: https://archive.org/details/volume-6-issue-6-november-december-2020/MC1_OA_Megha20edit/

[B53] Seligman M. (2011). Flourish. New York, NY: Free Press

[B54] Selin H. Davey G. (2012). Introduction. In Happiness Across Cultures. Science Across Cultures: The History of Non-Western Science, vol 6. Selin H. Davey G. (eds). Springer, Dordrecht. 10.1007/978-94-007-2700-7

[B55] Sharma P. Patra S. (2014). Exploring college student's conception of happiness. Indian J. Posit. Psychol. 5, 393.

[B56] Singh K. Jain A. Singh D. (2014a). Satsang: A culture specific effective practice for well-being. In Positive nations and communities. Springer. p. 79–100. 10.1007/978-94-007-6869-7_5

[B57] Singh K. Junnarkar M. Singh D. Suchday S. Mitra S. Dayal P. (2019). Associations between religious/spiritual practices and well-being in Indian elderly rural women. J. Relig. Health. 1–22. 10.1007/s10943-019-00877-9 31278629

[B58] Singh K. Kaur J. Singh D. (2014b). Well-being of rural women in North India. J. Indian Acad. Appl. Psychol. 40, 129. Availabe online at: https://www.researchgate.net/profile/Kamlesh-Singh-6/publication/299366330_Well-_being_of_Rural_Women_in_North_India/links/608b6ad9458515d315e6be03/Well-being-of-Rural-Women-in-North-India.pdf

[B59] Sotgiu I. (2016). Conceptions of happiness and unhappiness among Italian psychology undergraduates. PLoS ONE. 11, Article e0167745. 10.1371/journal.pone.0167745 27977718 PMC5157991

[B60] Srivastava A. Shukla A. (2018). Subjective happiness and differential loneliness among Indian adults. Int. J. Humanit. Arts Soc. 4, 1–10. 10.26855/jhass.2017.04.001

[B61] Suar D. Jha A. K. Das S. S. Alat P. Patnaik P. (2020). What Do Millennials Think of Their Past, Present, and Future Happiness, and Where Does Their Happiness Reside? J. Constructivist Psychol. 1–17. 10.1080/10720537.2020.1805657

[B62] Sutton C. (2021). What counts as happiness for young people: A qualitative study. Children Society. 35, 18–33. 10.1111/chso.12387

[B63] Trinh L. Khanh H. (2019). Happy people: Who are they? A pilot indigenous study on conceptualization of happiness in Vietnam. Health Psychol Rep. 7, 296–304. 10.5114/hpr.2019.88527

[B64] Tse S. (2017). Wellbeing in non-western cultures. In Slade M. Oades L. Jarden A. (Eds.), Wellbeing, recovery and mental health. Cambridge University Press. p. 194–205. 10.1017/9781316339275.017

[B65] Uchida Y. Kitayama S. (2009). Happiness and unhappiness in east and west: themes and variations. Emotion. 9, 441. 10.1037/a0015634 19653765

[B66] Uchida Y. Norasakkunkit V. Kitayama S. (2004). Cultural constructions of happiness: theory and emprical evidence. J. Happiness. Stud. 5, 223–239. 10.1007/s10902-004-8785-9

[B67] Uchida Y. Yuji O. (2012). Personal or interpersonal construal of happiness: A cultural psychological perspective. Int J Wellbeing. 2. 10.5502/ijw.v2.i4.58486778

[B68] Veenhoven R. (2000). Freedom and happiness: A comparative study in forty-four nations in the early 1990s. Culture and Subjective Wellbeing. 257–288. https://psycnet.apa.org/record/2000-16279-009

[B69] Veenhoven R. (2015). Social conditions for human happiness: A review of research. Int J Psychol. 50, 379–391. 10.1002/ijop.12161 25899153

[B70] Wani M. Dar A. A. (2017). Optimism, happiness, and self-esteem among university students. Indian J. Posit. Psychol. 8, 275–279. 26774001

[B71] Wills-Herrera E. Islam G. Hamilton M. (2009). Subjective well-being in cities: A multidimensional concept of individual, social and cultural variable. Appl. Res. Qual. Life. 4, 201–221. 10.1007/s11482-009-9072-z

[B72] Ye D. Ng Y.-K. Lian Y. (2015). Culture and happiness. Soc. Indic. Res. 123, 519–547. 10.1007/s11205-014-0747-y 26300581 PMC4541708

[B73] Zhang J. W. Howell R. T. Iyer R. (2014). Engagement with natural beauty moderates the positive relation between connectedness with nature and psychological well-being. J Environ,. Psychol. 38, 55–63. 10.1016/j.jenvp.2013.12.013

